# Unifying networks of a rhythm

**DOI:** 10.7554/eLife.104698

**Published:** 2024-11-28

**Authors:** Mohsen Alavash

**Affiliations:** 1 Center of Brain, Behavior and Metabolism, University of Lübeck Lübeck Germany

**Keywords:** dopamine, beta oscillations, invasive electrophysiology, brain connectivity, neural circuits, Human

## Abstract

Combining electrophysiological, anatomical and functional brain maps reveals networks of beta neural activity that align with dopamine uptake.

**Related research article** Chikermane M, Weerdmeester L, Rajamani N, Köhler RM, Merk T, Vanhoecke J, Horn A, Neumann WJ. 2024. Cortical beta oscillations map to shared brain networks modulated by dopamine. *eLife* **13**:RP97184. doi: 10.7554/eLife.97184.

Our brains constantly receive and respond to information from the world around us. This ability largely relies on communication between different areas of the brain via networks of neurons, as well as the release of molecules known as neurotransmitters. The interplay between neural networks and neurotransmitters manifests in waxing and waning patterns of electrical activity, known as brain rhythms, which oscillate at different frequencies.

However, understanding how areas of the brain communicate with one another has remained a major challenge in neuroscience, partly because directly measuring the activity of neurons in the cortex requires invasive procedures. This means that such measurements are typically only possible in animals or in patients undergoing surgery, and are usually limited to a few localized sites in the cortex (Frauscher et al., 2018).

Additionally, mapping how different brain regions connect and communicate requires various imaging techniques. Magnetic resonance imaging (MRI) is needed to track how regions are anatomically and functionally linked, while positron emission tomography (PET) is required to observe the uptake of neurotransmitters. However, integrating these brain imaging data has proven challenging (Hansen et al., 2022). Now, in eLife, Wolf-Julian Neumann and colleagues from Charité-Universitätsmedizin Berlin and Humboldt University – including Meera Chikermane as first author – report an integrative approach that provides insights into an important rhythmic pattern of neural activity known as the beta rhythm (Chikermane et al., 2024).

Beta patterns of neural activity oscillate within a frequency band between 13–35 Hz, and are critical for controlling movements, making decisions, and regulating emotions (Spitzer and Haegens, 2017; Lundqvist et al., 2024). In Parkinson’s disease, aberrant beta activity has been associated with less neurons deep in the brain releasing dopamine, a key neurotransmitter involved in synaptic plasticity (and therefore learning), as well as regulating movement and emotions (Cavallo and Neumann, 2024). This led Chikermane et al. to hypothesize that beta-band brain networks would align with the distribution of dopamine-sensitive receptors.

To test their hypothesis, the team adopted an innovative approach (based on multimodal lesion network mapping [Fox, 2018]) that integrates different types of imaging data and maps within a shared standard brain mapping framework. This allows the different types of data to be compared with one another. First, they used electrophysiological recordings taken from healthy cortical areas in 106 epileptic patients to identify brain regions that exhibited strong beta oscillations (Figure 1, yellow node).

**Figure 1.**
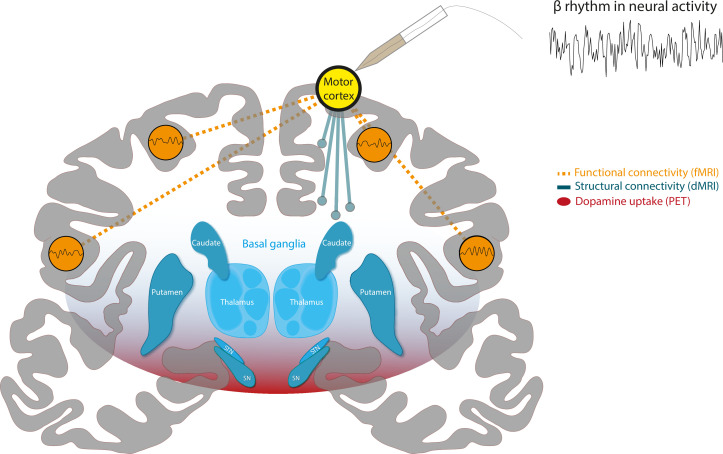
A combination of different imaging techniques can be used to unify brain networks associated with beta rhythms in neural activity. Schematic of a coronal section of the human brain, with the cortex in gray and a structure deep within the brain known as the basal ganglia in blue. Surface electrodes were used to record neural activity from epileptic patients undergoing surgery. This revealed which areas of the brain display rhythmic patterns of activity within the beta frequency range (13–35 Hz), represented as a yellow circle over the motor cortex. Functional MRI (fMRI) data (measurements of blood oxygen level) revealed the brain areas that showed synchronized changes in activity (orange dashed lines) with target regions. This suggests that there is a functional network of beta regions (orange circles). Diffusion-weighted MRI (DW-MRI) data showed that the target regions also connect to other parts of the brain through white matter tracts, forming a structural network (blue solid lines). Finally, positron emission topography (PET) data was used to identify brain regions with high dopamine uptake. Dopamine signaling in these areas was found to align with the connectivity of beta networks (red shading).

Image credit: Adapted from SciDraw (CC BY 4.0).

Chikermane et al. designated these sites as target regions, and then employed open access MRI data to build network maps, which were derived from two types of MRI data: functional MRI (fMRI), which measures blood oxygen level and can be used to determine which parts of the brain are synchronously activated (Figure 1, dashed connections), and diffusion-weighted MRI (DW-MRI), which shows how different regions of the brain are structurally connected by bundles of nerve tissues known as white matter tracts (Figure 1, solid connections). Additionally, PET imaging data, which relies on intravenous injections of a radioactively labelled molecule, were used to map which areas of the brain have a high uptake of dopamine, suggesting there is a high density of dopamine receptors (Figure 1, red shading). The three network maps – constructed using the fMRI, DW-MRI and PET data – were then integrated using a software known as the Lead-DBS toolbox.

The results revealed that beta activity is widespread throughout the brain. In fact, out of 1,772 surface electrodes placed in the cortex of the patients studied, only 21 did not exhibit beta oscillations. Among these cortical sites, beta signals were strongest across the frontal, cingulate, and insular regions of the brain. Importantly, all three of these areas map onto regions that were shown to be both functionally and structurally connected. This shared network also positively correlated with sites in the cortex and basal ganglia where high levels of dopamine receptor binding were observed. Collectivity, these findings help unify networks associated with beta rhythms in the human brain.

Chikermane et al. have circumvented the limitations of each electrophysiology and brain imaging technique in a synergistic way. Their results represent a significant step forward in mapping the organization of brain regions that display beta activity. The findings have important implications for understanding the circuit-level pathophysiology associated with altered beta activity, and how dopamine contributes to movement disorders such as Parkinson’s disease. Additionally, the findings suggests that improvements to deep brain stimulation, a technique that uses electrical pulses to stimulate structures deep in the brain, could potentially enhance motor function in Parkinson’s disease patients. However, whether and how the brain mapping technique developed by Chikermane et al. can be used to characterize and alleviate individual patient symptoms, or predict responsiveness to interventions, remains an open question for future research.
